# Mediating role of Social Support and Self-efficiency on Academic stress and Student’s Psychological well-being among Public and Private Universities in Mogadishu -Somalia.

**DOI:** 10.12688/f1000research.155275.1

**Published:** 2024-09-30

**Authors:** Ali Dahir Mohamed, Abdulkadir Jeilani

**Affiliations:** 1 Faculty of Education, Mogadishu University, Mogadishu University, Somalia; 2 Faculty of Computer Science & IT, Mogadishu University, Mogadishu, Somalia

**Keywords:** Social Support, Self-efficiency, Academic stress, psychological well-being, Public and Private Universities

## Abstract

**Background:**

Academic stress is a significant factor affecting students’ psychological well-being, particularly in higher education. Understanding the mediating roles of social support and self-efficacy can provide insights into how these factors influence students’ psychological well-being in public and private universities.

**Methods:**

The study examined the mediating roles of social support and self-efficacy in the relationship between academic stress and psychological well-being among university students in Mogadishu, Somalia. Utilizing a stratified sampling technique, data were collected from 663 students across public (52.6%) and private (47.4%) universities through a structured questionnaire and analyzed using path analysis to assess direct and indirect effects. Structural equation modeling technique was used for data analysis.

**Results:**

The analysis revealed a significant direct effect of academic stress on psychological well-being (β = 0.133, p = 0.000). Academic stress was not significantly related to social support (β = 0.128, p = 0.066) and self-efficacy (β = 0.075, p = 0.099). However, both social support (β = 0.059, p = 0.000) and self-efficacy (β = 0.838, p = 0.000) were significantly related to psychological well-being. The mediating analysis showed that social support partially mediated the relationship between academic stress and psychological well-being (Indirect effect = 0.070, CI [-0.036, 0.197], p = 0.000), while self-efficacy did not mediate this relationship (Indirect effect = 0.063, CI [0.054, 0.218], p = 0.097).

**Conclusions:**

The findings suggest a significant positive impact of academic stress on psychological well-being, while social support partially mediates this relationship, highlighting its buffering role. Conversely, self-efficacy, although positively contributing to psychological well-being, does not mediate the effect of academic stress. These results underscore the importance of robust social support systems and targeted interventions to enhance students’ coping mechanisms and overall psychological well-being. Study limitations and implication are discussed.

## 1. Introduction

Stress is one of the most important factors in psychological research since it has an impact on the well-being and health of individuals, the psychological well-being of students is significantly harmed by academic stress, which is caused by work, high performance, tests, and challenging assignments. Therefore, it is essential to fully understand the complex relationship between academic stress and psychological well-being in order to develop effective treatments. ^1^

Academic stress refers to the stress experienced by individuals in educational settings due to numerous variables, According to earlier research ^2^^–^^5^ Academic stress arises from the inability to cope with academic related tasks, leading to negative impacts such as smoking behavior, mental health issues, poor sleep quality, depression, insomnia, substance addiction, self-harm, and suicidal ideation. Also, other factors contributing to academic stress include self-inflicted stress, parental expectations, academic queries, lack of time for revision, low parental education levels, and poor exam grades. Therefore, understanding academic stress is crucial for implementing interventions at various levels to mitigate its negative effects on students’ well-being and academic performance.

Academic stress is the product of a combination of academic-related demands that exceed the adaptive resources available to an individual, ^2^ Researchers agree that students face common academic stressors, such as family-pressures, scholarship demands, financial burdens, competition in class, examination, time-management and course-related stress. ^2^ According to Ref. 3 the main cause of academic stress are academic results, admissions, students transfer, exams, load of extra classes, social comparison, exams stress, expectations teachers and parents.

Academic stress has been shown to have negative impact on students’ mental health, Researches ^6^ indicates that high levels of academic stress are correlated with an increased probability of experiencing mental health issues, such as weakening mental health and feelings of anxiety and depression. ^5^ Studies have highlighted the importance of addressing academic stress to enhance students’ overall mental well-being, as stress from academic pressures, family circumstances, and other sources can significantly impact mental health. Furthermore, interventions like wellness trainings have shown promise in improving mental wellness and reducing the negative impact of academic stress on students’ mental health.

The most common psychological consequences associated with academic stress include depression, anxiety, substance abuse, and suicide ideation. ^7^ Higher stress levels, particularly related to academic tasks and concerns like grades, exams, and competition with peers, are predominant among younger students. ^8^ These psychological consequences highlight the importance of addressing and managing academic stress effectively to safeguard students’ mental health and well-being.

Academic stress significantly impacts the psychological well-being of students in both public and private institutions. Studies show that academic stress is associated with lower mental well-being. ^9^^,^^10^ Factors such as academic pressure, family circumstances, side-activity pressure, and financial situation contribute to increased stress levels among students, leading to a negative impact on mental well-being. Additionally, coping strategies like Academic Psychological capital and self-efficacy play a crucial role in mitigating the effects of academic stress on psychological well-being. ^11^ It is essential for educational institutions to develop strategies to reduce stress levels and support students in managing academic pressures to safeguard their psychological well-being.

Researchers have identified protective variables that may protect students from the negative psychological effects of study-related stress. But most research has focused on identifying the psychological symptoms of academic stress, rather than looking at strategies to protect students from these demands. ^12^

In both public and private universities in Mogadishu, Somalia, the current study aimed to examine the effects of academic stress on students’ psychological wellbeing, with an emphasis on the mediating roles of self-efficiency and social support. By integrating the Transactional Theory of Stress and Coping with the Social Cognitive Theory, researchers can gain a broader understanding of how academic stress impacts students’ psychological well-being, especially in the context of social support and self-efficacy, across public and private universities. These theories provide a framework for exploring the complex interactions among stress, coping, social support, and self-efficacy in shaping students’ well-being outcomes in academic settings. The study addressed the absence of comprehensive research on the impacts of academic stress on students’ psychological well-being in both public and private universities.

## 2. Literature review

### 2.1 Transactional theory and of stress coping

The Transactional Model of Stress and Coping, proposed by Lazarus and Folk man ^13^ (1984), contended that a person’s capacity to cope and adjust to challenges and problems is a consequence of transactions (or interactions) that occur between a person and their environment. The Transactional Theory of Stress and Coping, developed by Lazarus and Folk man, focuses on how individuals assess and response to stress through cognitive appraisal and cognitive processes. stress is the result of transactions between the person, environment, and situation, influenced by cognitive appraisals and coping strategies. This theory emphasizes the dynamic nature of stress and coping processes, and suggests that individuals’ perceptions and responses to stressors play an important role in determining their well-being outcomes ^13^^,^^14^

### 2.2 Social Cognitive Theory

The Social Cognitive Theory, proposed by Albert Bandura, emphasizes the role of cognitive processes in shaping behavior and outcomes. this means that persons’ beliefs about their ability to succeed (self-efficacy) influence their behavior and reactions to challenging situations. In the context of academic stress, this theory suggests that the students have the ability to cope with high self-efficacy are more likely to effectively cope with stressors and maintain their psychological well-being. Social Cognitive Theory also considers the impact of social influences and observational learning on individuals’ coping. ^9^

In the context of the impact of academic stress on students’ psychological well-being, the Transactional Theory of Stress and Coping and the Social Cognitive Theory focus on the following. Both theories highlight the importance of cognitive appraisal in how individuals perceive and respond to stress. Students’ cognitive evaluations of academic stressors, their perceived ability to cope (self-efficacy), and the availability of social support influence their stress experiences and psychological well-being. ^10^

In Coping Mechanisms, the theories emphasize the importance of coping strategies in managing stress. Student performance related to cognitive appraisals and self-efficacy beliefs play an important role in mediating the relationship between academic stress and psychological well-being. Effective strategies, supported by social resources, help students manage academic stress and maintain well-being. ^9^^,^^10^^,^^15^

### 2.3 Academic Stress and Psychological Well-being

Academic stress has become a major mental health concern across the worldwide these days. Also has emerged as a significant mental health that affecting various groups of students. Studies ^4^^,^^8^ have highlighted the detrimental impact of academic stress on mental well-being, with associations found between high levels of academic stress and issues like depression, anxiety, self-harm, and suicidal ideation.

Academic stress among university students has been extensively studied, revealing various insights. According to research, there is a strong link between mental health and academic stress ^4^ with self-inflicted stress being a prominent factor in high stress levels among students. Critical thinking skills were found not to have a significant relationship with academic stress. ^12^ However, academic stress was positively correlated with academic performance, motivating students to study harder. ^16^ Academic stress significantly impacts the mental well-being of university students. ^1^^,^^14^^,^^16^ High levels of academic stress have been linked to increased probability of experiencing mental health issues like Weakening Mental Health. ^13^

A study found a strong correlation (r=0.582) between academic stress and psychological well-being among university students, indicating that higher stress levels correspond to lower well-being. ^17^

### 2.4 Mediating Role of Social Support

Social support plays a crucial role in mediating academic stress and enhancing students’ psychological well-being. Studies ^18^^–^^21^ indicated that social support from various sources like family, friends, and significant others positively correlates with happiness, subjective well-being, satisfaction with life, and flourishing among university students.

The impact of social support on students’ levels of academic stress is significant. Studies reveal a negative relationship between academic stress and parental social support, with more encouragement resulting into reduced stress levels. ^22^ Additionally, perceived social support from various sources like family, friends, and significant others has been found to significantly correlate with reduced stress levels among undergraduate Health Sciences students. ^17^ Moreover, teacher support at the individual level is associated with better coping abilities and lower helplessness, while peer support at the class level is linked to enhanced coping abilities and academic achievement. ^23^ Furthermore, peer social support has been shown to lower academic stress levels during online learning, highlighting its importance in helping students deal with the challenges of virtual education. ^24^^,^^25^

### 2.5 Mediating role of self-efficacy

Self-efficacy plays an important role in mediating the relationship between academic stress and students’ psychological well-being. Studies have shown ^26^^–^^29^ that higher levels of academic self-efficacy are associated with greater well-being, while academic stress tends to lead to psychological distress, such as symptoms of anxiety and depression. Additionally, factors like social support and mindfulness have been identified as mediating variables that influence academic self-efficacy in the face of psychological distress, ultimately impacting students’ well-being.

Self-efficacy plays an essential role in influencing academic performance across various educational settings. Research indicates that self-efficacy positively impacts academic achievement. ^30^^–^^33^ It acts as a predictor of academic success and can mitigate academic burnout, reduce dropout rates, and enhance engagement. ^18^ Moreover, self-efficacy mediates the relationship between parental expectations, academic stress, and performance, emphasizing its significance in academic outcomes. The correlation between self-efficacy and academic performance is particularly evident in vocational high school students, where self-efficacy significantly influences academic success. Overall, enhancing self-efficacy levels is vital for improving academic performance and student outcomes in various educational domains, highlighting the importance of fostering self-belief and confidence in learners. Therefore, the need to study the relationship between Academic stress on Student’s Psychological well-being, the following hypothesis is suggested.

Based on the above discussion the researchers proposed the following hypotheses:

H1.
There is a significant impact of academic stress on psychological well-being,

H2.
There is a significant impact of academic stress on social support.

H3.
There is a significant impact of academic stress on self-efficiency.

H4.
There is a significant impact of social support on psychological well-being,

H5.
There is a significant impact of self-efficiency on psychological well-being.

H6.
Social supports mediate the relationship between academic stress and psychological well-being.

**Figure 1.**
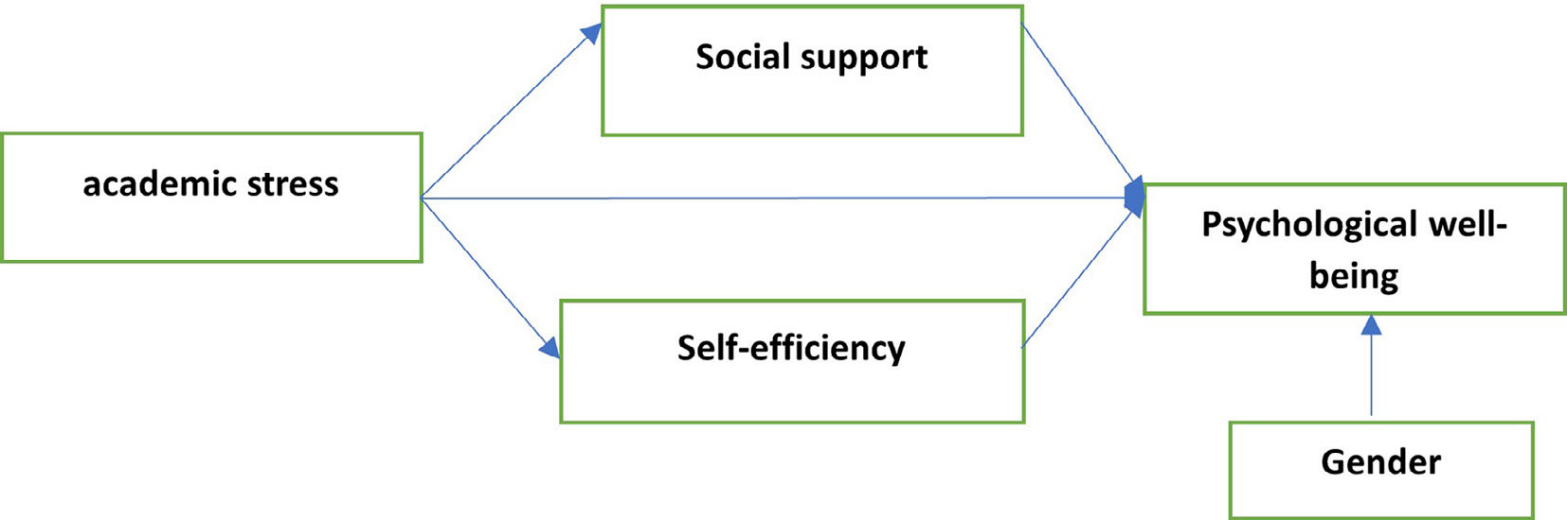
Research Model proposed by the authors

H7.
Self-efficiency mediate the relationship between academic stress and psychological well-being ( Figure 1).

## 3. Methods

### 3.1 Context of the study, population and sample

The context of the study was public and private universities in Mogadishu – Somalia. Nearly 50,000 students are intended in these universities. These universities have fields of study at undergraduate, diploma and postgraduate levels. Faculties provided these universities include social science, computer technology, public health, business Economics and management sciences, sharia and law studies, education, engineering, medicine and surgery, Arts & humanities, agriculture & Environmental Sciences, languages, Islamic studies, geology, media & journalism, public admin and politics dentistry and postgraduate studies. The population of the study are target population from which the sample is actually selected or aggregation of study elements. ^19^ The study population are undergraduate students of these universities, its estimated around 30,000 students. The study included stratified sample technique. Stratified sample technique is type of sampling method in which the total population is divided into smaller groups or strata to complete the sampling process. ^20^ The study examined various factors related to academic stress and psychological well-being. The sample includes 663 students, with a gender distribution of 40.4% female and 59.6% male.

### 3.2 Research instrument and measures

A survey questionnaire was developed as the instrument for data collection. Items used for Academic Stress adapted by Ref. 21 to get students’ views of academic pressures and consists of 10 items, Psychological well-being Scale (PWS) adapted by Ref. 22 and consist of 18 items, Social Support Scale (SSS) adapted by Ref. 34 and 12 items, Self-Efficiency Scale (SSS) adapted by Ref. 35 and consist of 10 items.

### 3.3 Data collection strategy

Data collected using adopted questionnaires distributed electronically, depending on the feasibility and preferences of participants. The questionnaire comprised validated measures assessing academic stress, psychological well-being, social support, and self-efficacy. Additionally, demographic information such as age, gender, academic major, and university affiliation collected to facilitate subgroup analyses. The data collected from May 15 to Jun 3 2024.

### 3.4 Data analysis

Descriptive statistics used to characterize the sample and examine the distribution of key variables. Structural equation modeling was used to examine mediating analysis of social support, self-efficacy in the relationship between academic stress, and psychological well-being.

### 3.5 Ethical consideration

Prior to data collection, ethical approval was received from Mogadishu University institutional review board (IRB) in Mogadishu, Somalia (Date:15 – 05 – 2024, protocol number: MUIRB/09/24/001). Informed consent was obtained from all students. The students were informed that the research would be conducted following the principle of confidentiality and that all information obtained would be kept confidential.

## 4. Data analysis and results

### 4.1 Profile data

A total of 663 sample size was achieved, the demographic profile of respondents for this study showed that female respondents made up 40.4%, of the sample while male made up 59.6% and mostly aged 18 to 21 years 67.1%. The distribution between public (52.6%) and private (47.4%) universities is nearly even. Most students are in their first three years of study, with Year One students representing the largest group 36.2%, while there are few students in Year Five (0.9%) and Year Six (1.2%) ( Table 1).

**Table 1.** Demographic profile data.

		Frequency	Percentage (%)
Gender	Female	268	40.4%
	Male	395	59.6%
Age	18 to 21 years	445	67.1%
	22 to 31 years	212	32.0%
	32 to 41 years	6	0.9%
University sector	Public	349	52.6%
	Private	314	47.4%
Study year	Year One	240	36.2%
	Year Two	152	22.9%
	Year Three	178	26.8%
	Year Four	79	11.9%
	Year Five	6	0.9%
	Year Six	8	1.2%

Note: Primary data collected by authors 2024.

**Measurement model**

The factor loadings for the items under each construct are all above 0.50, indicating good convergent validity. ^36^ The composite reliability (CR) values for all constructs are above the recommended threshold of 0.70, suggesting good internal consistency. However, the AVE values for the academic stress (0.399) and psychological well-being (0.487) constructs are below the recommended threshold of 0.50, indicating that these constructs may not be capturing a sufficient amount of variance in their respective indicators. The correlation matrix reveals moderate to strong relationships between the constructs, with the highest correlation observed between self-efficacy factor and psychological well-being (0.814), suggesting a strong positive association between these two variables. Overall, the results provide insights into the psychometric properties of the measured constructs and their interrelationships within the study context. The discriminant validity was achieved ( Tables 2 and 3).

**Table 2.** Factor loading, Composite reliability (CR) and average variance extracted (AVE).

	Factor loading	Alpha	Composite reliability	AVE
Academic stress		0.755	0.768	0.399
AS1	0.604			
AS2	0.704			
AS3	0.614			
AS4	0.597			
AS5	0.632			
Social support factor		0.906	0.906	0.703
SO1	0.851			
SO2	0.855			
SO3	0.835			
SO4	0.819			
Self-efficacy factor		0.843	0.843	0.574
SEF1	0.787			
SEF2	0.790			
SEF3	0.706			
SEF4	0.744			
Psychology well-being		0.821	0.825	0.487
PWB1	0.740			
PWB2	0.782			
PWB3	0.595			
PWB4	0.674			
PWB5	0.683			

Note: Primary data collected by authors 2024.

**Table 3.** Discriminant validity.

	CR	AVE	SEF	AS	SSF	PWB
**SEF**	0.843	0.574	**0.758**			
**AS**	0.768	0.399	0.068	**0.631**		
**SSF**	0.906	0.706	0.516	0.076	**0.840**	
**PWB**	0.825	0.487	0.814	0.189	0.430	**0.698**

Note: Primary data collected by authors 2024.

**Structural model**

The research findings H1. show that academic stress significantly impacts students’ psychological well-being, with a path coefficient ( *β*) of 0.133, a *t*-statistic of 3.524, and a *p*-value of 0.000, indicating a strong positive relationship. However, academic stress does not significantly affect social support ( *β* = 0.128, *t* = 1.839, p = 0.066) or self-efficacy ( *β* = 0.075, *t* = 1.652, *p* = 0.099). Social support has a positive and significant effect on psychological well-being ( *β* = 0.059, *t* = 2.624, *p* = 0.000), suggesting that increased support is associated with better psychological outcomes. Self-efficacy has a very strong and significant positive impact on psychological well-being ( *β* = 0.838, *t* = 14.002, *p* = 0.000), highlighting its crucial role in enhancing students’ psychological health in the context of academic stress. The result presents in the Table 4.

**Table 4.** Hypotheses test.

	Path coefficient	T statistics	P values
H1: ASàPWB	0.133	3.524	0.000
H2: ASàSSF	0.128	1.839	0.066
H3: ASàSEF	0.075	1.652	0.099
H4: SSàPWB	0.059	2.624	0.000
H5: SEFàPWB	0.838	14.002	0.000

Note: Primary data collected by authors 2024.

**Mediation analysis**

The study assessed the mediating role of social support factor (SSF) and self–efficiency (SEF) on the relationship between academic stress (AS) and psychological well-being (PWB). The results revealed significant indirect effect of AS on PWB through SSF ( β=0.0075,t=5.2447,p<0.001), supporting H1. Analyzing the mediating role of SEF, the study found insignificant mediating role of SEF on the linkage between AS and PWB ( β=0.068,t=1.654,p=0.097), not supporting H2. Furthermore, the direct effect of AS on PWB in presence of the mediation was also found significant ( β=0.133,t=3.524,p<0.001). Hence, SSF partially mediated the relationship between AS and PWB. Mediation analysis summary is presented in Table 5.

**Table 5.** Mediating role social support and self-efficiency.

Relationship	Direct effect	Indirect effect	Confidence interval		P-value	Conclusion
			Lower Bounded	Upper Bound		
AS->SSF->PWB	.127	0.070	−0.036	0.197	0.000	Partially Mediation
AS->SEF->PWB	0.000	0.063	0.054	0.218	0.097	No Mediation

Note: Primary data collected by authors 2024.

**Figure 2.**
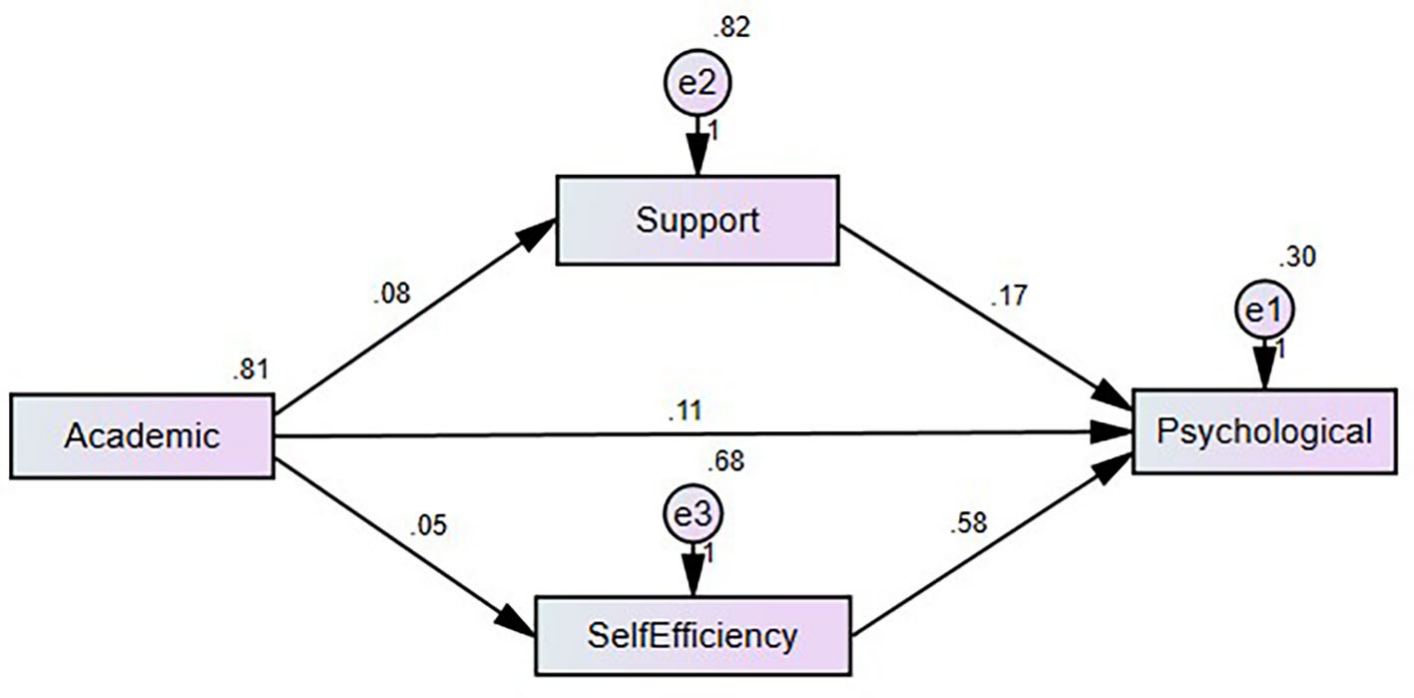
Path diagram showing the mediation model proposed by the authors

**Table 6.** Summary of the hypotheses.

Hypotheses	Finding
**H1.** There is a significant impact of academic stress on psychological well-being	Supported
**H2.** There is a significant impact of academic stress on social support.	Not supported
**H3.** There is a significant impact of academic stress on self-efficiency.	Supported
**H4.** There is a significant impact of social support on psychological well-being.	Supported
**H5.** Social supports mediate the relationship between academic stress and psychological well – being.	Partially Mediation
**H6.** Self-efficiency mediate the relationship between academic stress and psychological well-being.	No Mediation

Note: Primary data collected by authors 2024.

**Evaluation model fit**

The model evaluation results indicate a good fit of the model to the data. The relative chi-square (2.537) is within the recommended threshold of less than 5, and all the fit indices, including the Goodness of Fit Index (0.948), Adjusted Goodness of Fit Index (0.931), Comparative Fit Index (0.962), Incremental Fit Index (0.963), Normed Fit Index (0.940), Tucker-Lewis Index (0.955), and the Root Mean Square Error of Approximation (0.048), meet or exceed their respective recommended thresholds. Additionally, the Standardized Root Mean Square Residual (0.0369) is within the recommended limit of less than 0.05, ^26^ further supporting the good fit of the model ( Table 7).

**Table 7.** Model fit indices.

	Recommended	Result
Relative Chi-Sq	Chi<5	2.537
Goodness fit index (GFI)	GFI≥.90	0.948
Adjusted goodness fit index (AGFI)	AGFI≥.90	0.931
Comparative fit index (CFI)	CFI≥.90	0.962
Incremental fit index (IFI)	IFI≥.90	0.963
Normed fit index (NFI)	NFI≥.90	0.940
Tucker-Lewis Index	TFI≥.90	0.955
Root Mean Square Error of Approximation (RMSEA)	RMSEA≤0.08	0.048
Standard RMR	SRMR≤0.05	0.0369

Note: Primary data collected by authors 2024.

## 5. Discussion

The study is set out to establish the relationship between Academic stress (AS), Student’s Psychological well-being, social (PWB), Social support (SS) and Self-efficiency. The study found a significant impact of academic stress on student psychological well-being among public and universities in Mogadishu - Somalia. The outcomes affirm this recommendation are steady with the discoveries of the past examinations where the positive relationship between AS and PWB outcomes was found. ^27^^–^^29^ This relationship indicates that the pressures of academic demands, such as exams and assignments, can lead to negative psychological outcomes, including anxiety and decreased life satisfaction. The findings align with the Transactional Model of Stress and Coping, which suggests that stress occurs when individuals perceive demands exceeding their coping abilities. The unique socio-cultural and educational challenges in Somalia, such as limited resources and high expectations, may intensify the effects of academic stress, emphasizing the need for support systems and interventions to help students manage stress and improve their psychological well-being.

The study found insignificant impact of academic stress on social support factor, suggesting that academic stress does not significantly alter the level of social support students perceive or receive. This result may indicate that social support, whether from peers, family, or faculty, remains stable despite fluctuations in academic stress levels. Previous research has shown that while social support can buffer the effects of stress, the presence of stress does not necessarily reduce the availability or perception of social support. ^27^^–^^29^

This stability might be due to cultural or social norms that encourage strong support networks, regardless of individual stress levels. Additionally, social support might be more influenced by factors such as personal relationships and community dynamics rather than academic pressures.

The study found that academic stress had an insignificant impact on self-efficacy among students in public and private universities in Mogadishu. This suggests that the students’ belief in their ability to manage and succeed in academic tasks was not significantly affected by the levels of academic stress they experienced. This finding is inconsistent with research indicating that self-efficacy is often a stable trait influenced more by long-term experiences and personal characteristics than by situational stress. ^30^^,^^31^ Self-efficacy may be bolstered by intrinsic factors such as past successes, individual resilience, and consistent encouragement from mentors and peers, which help maintain students’ confidence in their abilities despite academic pressures.

The study found that social support significantly impacts psychological well-being (PWB) among students in public and private universities in Mogadishu. This means that students who receive higher levels of social support from peers, family, and faculty tend to have better psychological well-being, experiencing less anxiety and higher life satisfaction. This finding aligns with previous research which indicates that social support acts as a buffer against stress and promotes mental health by providing emotional comfort, practical assistance, and a sense of belonging. ^32^^,^^33^^,^^37^^,^^38^ The presence of a strong support network can mitigate the adverse effects of stress and enhance overall well-being by improving coping mechanisms and providing resources for managing academic challenges.

The study found that self-efficacy significantly impacts psychological well-being (PWB) among students in public and private universities in Mogadishu. This indicates that students who have a strong belief in their ability to manage and succeed in academic tasks tend to have better psychological well-being, with lower levels of anxiety and higher life satisfaction. This finding is supported by previous research showing that self-efficacy enhances mental health by promoting resilience, reducing stress, and encouraging proactive coping strategies. ^37^^,^^39^^,^^40^ High self-efficacy helps students to perceive challenges as manageable and to maintain a positive outlook, which contributes to better psychological well-being.

Finally, the results of this research offer important empirical insight into the indirect effect on academic stress on psychological well-being through mediation of social support factor. The finding shows that social support partially mediation the relationship between academic stress and psychological well-being. The findings are consistent with the previous finding which have found the significant mediating role of social support, ^33^^,^^41^ which suggests that social support can mitigate the harmful effects of stress by providing emotional comfort, practical help, and a sense of belonging, thus enhancing overall psychological well-being. On other hand, the study found that self-efficacy does not mediate the relationship between academic stress and psychological well-being (PWB) among students in Mogadishu’s universities. This means that while self-efficacy directly contributes to better psychological well-being, it does not significantly alter the impact of academic stress on well-being. This finding contrasts with some studies that suggest self-efficacy can buffer stress impacts, ^33^ indicating that in this context, other factors may play a more significant role in mediating the relationship between academic stress and psychological well-being.

## 6. Conclusion

In conclusion, the study reveals several key findings about the relationships between academic stress, psychological well-being (PWB), social support, and self-efficacy among students in Mogadishu’s universities. Academic stress significantly impacts students’ psychological well-being, highlighting the need for interventions to manage academic pressures. Social support plays a critical role in partially mediating this relationship, buffering some of the negative effects of academic stress on mental health. In contrast, self-efficacy, while positively contributing to psychological well-being, does not mediate the relationship between academic stress and PWB. These insights underscore the importance of enhancing social support networks and developing targeted strategies to improve student resilience and mental health in the face of academic challenges.

### 6.1 Implication

Universities should prioritize the development and enhancement of student support services, including counseling and mental health resources, to help students manage academic stress and improve psychological well-being. More broadly, the study contributes to the development of studies linking academic stress, social support, self -efficacy and psychological well-being and strengthens the relationship evidence in the literature that social support contributes to psychological well -being. Institutions should encourage the formation of strong social networks through peer mentoring, student organizations, and community-building activities, as social support is crucial in buffering the negative effects of academic stress. Although self-efficacy does not mediate the stress-well-being relationship, enhancing students’ confidence in their abilities through workshops, positive feedback, and opportunities for skill development can directly improve their psychological well-being. Additionally, the study adds knowledge to studies seeking to understand the factors that influence psychological well-being. Thus, the findings suggest that universities should develop and implement stress management programs that equip students with strategies to cope with academic demands, thus improving their psychological well-being. Institutions should promote social support systems through peer mentoring, counseling services, and community-building activities to buffer students against the negative effects of academic stress. Institutions should promote social support systems through peer mentoring, counseling services, and community-building activities to buffer students against the negative effects of academic stress. Educational policies should prioritize mental health by incorporating stress management and support systems into the academic environment, ensuring holistic student development. Finally, it’s important that educational policymakers should integrate mental health education and stress management strategies into the curriculum, ensuring students are equipped with the tools to handle academic pressures effectively.

### 6.2 Research limitations and further Research

The study’s limitations include its cross-sectional design, which limits causal inference, and the reliance on self-reported measures, which may introduce biases. Additionally, the findings are context-specific to Mogadishu, Somalia, and may not be generalizable to other settings. The research also focused narrowly on social support and self-efficacy as mediators, potentially overlooking other influential factors. Future studies should explore other potential mediators and moderators in the relationship between academic stress and psychological well-being, such as coping styles, resilience, and environmental factors, to develop a more comprehensive understanding and targeted interventions.

#### Ethical consideration

Prior to data collection, ethical approval was received from Mogadishu University institutional review board (IRB) in Mogadishu, Somalia (Date:15 – 05 – 2024, protocol number: MUIRB/09/24/001). Informed consent was obtained from all students. The students were informed that the research would be conducted following the principle of confidentiality and that all information obtained would be kept confidential. We obtained informed consent from all participants, according to the IRB’s guidelines. Each of the 663 participants reviewed and signed the consent form before participating in the study. We confirm that all participants adhered to the signed consent form.

## Data Availability

Figshare: Mediating role of Social Support and Self-efficiency on Academic stress and Student’s Psychological well-being among Public and Private Universities in Mogadishu -Somalia. https://doi.org/10.6084/m9.figshare.26820745 ^42^ The project contains the following data:
- Academic stress and Psychological well being.xlsx - Pschological Well being Mediating Role Updated.sav Figshare: Mediating role of Social Support and Self-efficiency on Academic stress and Student’s Psychological well-being among Public and Private Universities in Mogadishu-Somalia. https://doi.org/10.6084/m9.figshare.26820745 ^42^ The project contains the following data:
- Analyzing the results and calculating p-value using 5000 Bootstrap.docx - Measurement model.jpg Data are available under the terms of the Creative Commons Zero “No rights reserved” data waiver (CC0 1.0 Public domain dedication).
