# Mediating role of Social Support and Self-efficacy on Academic stress and Student’s Psychological well-being among University Students in Mogadishu -Somalia.

**DOI:** 10.12688/f1000research.155275.2

**Published:** 2025-02-13

**Authors:** Ali Dahir Mohamed, Abdulkadir Jeilani

**Affiliations:** 1 Faculty of Education, Mogadishu University, Mogadishu University, Somalia; 2 Faculty of Computer Science & IT, Mogadishu University, Mogadishu, Somalia

**Keywords:** Social Support, Self-efficiency, Academic stress, psychological well-being, Public and Private Universities

## Abstract

**Background:**

Academic stress is a significant factor affecting students’ psychological well-being, particularly in higher education. Understanding the mediating roles of social support and self-efficacy can provide insights into how these factors influence students’ psychological well-being in public and private universities.

**Methods:**

The study examined the mediating roles of social support and self-efficacy in the relationship between academic stress and psychological well-being among university students in Mogadishu, Somalia. Utilizing a stratified sampling technique, data were collected from 663 students across public (52.6%) and private (47.4%) universities through a structured questionnaire and analyzed using path analysis to assess direct and indirect effects. Structural equation modeling technique was used for data analysis.

**Results:**

The analysis revealed a significant direct effect of academic stress on psychological well-being (β = 0.087, p = 0.001). Social support was significantly related to Academic stress (β = 0.117, p = 0.031) and self-efficacy (β =0.088, p = 0.021). However, both social support (β = 0.099, p = 0.000) and self-efficacy (β = 0.646, p = 0.000) were significantly related to psychological well-being. The mediating analysis showed that social support partially mediated the relationship between academic stress and psychological well-being (Indirect effect = 0.012, CI [0.002, 0.024], p = 0.000), while self-efficacy did not mediate this relationship (Indirect effect = 0.057, CI [0.006, 0.111], p = 0.068).

**Conclusions:**

The findings suggest a significant positive impact of academic stress on psychological well-being, while social support partially mediates this relationship, highlighting its buffering role. Conversely, self-efficacy, although positively contributing to psychological well-being, does not mediate the effect of academic stress. These results underscore the importance of robust social support systems and targeted interventions to enhance students’ coping mechanisms and overall psychological well-being. Study limitations and implication are discussed.

## 1. Introduction

Stress is one of the most important factors in psychological research since it has an impact on the well-being and health of individuals, the psychological well-being of students is significantly harmed by academic stress, which is caused by work, high performance, tests, and challenging assignments. Therefore, it is essential to fully understand the complex relationship between academic stress and psychological well-being in order to develop effective treatments. ^1^

Academic stress refers to the pressure experienced by individuals in educational settings due to a range of factors, primarily arising from an inability to manage academic-related tasks effectively. This stress can lead to various negative consequences, such as smoking behavior, mental health issues, poor sleep quality, depression, insomnia, substance addiction, self-harm, and suicidal ideation. Contributing factors include self-inflicted stress, parental expectations, academic queries, lack of time for revision, low parental education levels, poor exam grades, family pressures, scholarship demands, financial burdens, classroom competition, time management challenges, and course-related stress. ^2^^–^^8^ Coping strategies such as academic psychological capital and self-efficacy can mitigate these effects. However, most research focuses on symptoms rather than protective strategies. Educational institutions should develop interventions to reduce stress and support students in managing academic pressures to protect their mental health. ^9^^–^^11^

The current study aimed to examine the effects of academic stress on students’ psychological wellbeing, with an emphasis on the mediating roles of self-efficacy and social support. By integrating the Transactional Theory of Stress and Coping with the Social Cognitive Theory, researchers can gain a broader understanding of how academic stress impacts students’ psychological well-being, especially in the context of social support and self-efficacy, across public and private universities. These theories provide a framework for exploring the complex interactions among stress, coping, social support, and self-efficacy in shaping students’ well-being outcomes in academic settings. The study addressed the absence of comprehensive research on the impacts of academic stress on students’ psychological well-being in both public and private universities.

## 2. Literature review

### 2.1 Transactional theory and of stress coping

The Transactional Model of Stress and Coping, proposed by Ref. 12, focuses on how individuals assess and response to stress through cognitive appraisal and cognitive processes. Stress is the result of transactions between the person, environment, and situation, influenced by cognitive appraisals and coping strategies. This theory emphasizes the dynamic nature of stress and coping processes, and suggests that individuals’ perceptions and responses to stressors play an important role in determining their well-being outcomes. ^13, 14^

### 2.2 Social Cognitive Theory

The Social Cognitive Theory, proposed by Ref. 15, emphasizes the role of cognitive processes in shaping behavior and outcomes. This means that persons’ beliefs about their ability to succeed (self-efficacy) influence their behavior and reactions to challenging situations. In the context of academic stress, this theory suggests that the students have the ability to cope with high self-efficacy are more likely to effectively cope with stressors and maintain their psychological well-being. Social Cognitive Theory also considers the impact of social influences and observational learning on individuals’ coping. ^16^

### 2.3 Cognitive Appraisal and Coping Mechanisms in Managing Academic Stress

The Transactional Theory of Stress and Coping and the Social Cognitive Theory emphasize the importance of cognitive appraisal in how students perceive and respond to academic stress. Factors such as students’ evaluations of stressors, self-efficacy, and social support significantly influence their stress experiences and overall psychological well-being. ^17^

In coping mechanism, the theories emphasize the importance of coping strategies in managing stress. Student performance related to cognitive appraisals and self-efficacy beliefs play an important role in mediating the relationship between academic stress and psychological well-being. Effective strategies, supported by social resources, help students manage academic stress and maintain well-being. ^9, 14, 18^

### 2.4 Academic Stress and Psychological Well-being

Academic stress has become a major mental health concern across the worldwide these days. Also has emerged as a significant mental health that affecting various groups of students. Studies, ^5, 8^ have highlighted the detrimental impact of academic stress on mental well-being, with associations found between high levels of academic stress and issues like depression, anxiety, self-harm, and suicidal ideation.

Academic stress among university students has been extensively studied, revealing various insights. According to research, there is a strong link between mental health and academic stress ^6^ with self-inflicted stress being a prominent factor in high stress levels among students. Critical thinking skills were found not to have a significant relationship with academic stress. ^11^ However, academic stress was positively correlated with academic performance, motivating students to study harder. ^19^ Academic stress significantly impacts the mental well-being of university students. ^3, 19, 20^ High levels of academic stress have been linked to increased probability of experiencing mental health issues like Weakening Mental Health. ^13^

A study found a strong correlation (r=0.582) between academic stress and psychological well-being among university students, indicating that higher stress levels correspond to lower well-being. ^16^

### 2.5 Mediating Role of Social Support

Social support plays a crucial role in mediating academic stress and enhancing students’ psychological well-being. Studies ^21, 22^ indicated that social support from various sources like family, friends, and significant others positively correlates with happiness, subjective well-being, satisfaction with life, and flourishing among university students.

The impact of social support on students’ levels of academic stress is significant. Studies reveal a negative relationship between academic stress and parental social support, with more encouragement resulting into reduced stress levels. ^22^ Additionally, perceived social support from various sources like family, friends, and significant others has been found to significantly correlate with reduced stress levels among undergraduate Health Sciences students. ^16^ Moreover, teacher support at the individual level is associated with better coping abilities and lower helplessness, while peer support at the class level is linked to enhanced coping abilities and academic achievement. ^23^ Furthermore, peer social support has been shown to lower academic stress levels during online learning, highlighting its importance in helping students deal with the challenges of virtual education. ^24, 25^

### 2.6 Mediating role of self-efficacy

Self-efficacy plays an important role in mediating the relationship between academic stress and students’ psychological well-being. Studies have shown ^25^^,^^26^ that higher levels of academic self-efficacy are associated with greater well-being, while academic stress tends to lead to psychological distress, such as symptoms of anxiety and depression. Additionally, factors like social support and mindfulness have been identified as mediating variables that influence academic self-efficacy in the face of psychological distress, ultimately impacting students’ well-being.

Self-efficacy plays an essential role in influencing academic performance across various educational settings. Research indicates that self-efficacy positively impacts academic achievement. ^27^ It acts as a predictor of academic success and can mitigate academic burnout, reduce dropout rates, and enhance engagement. ^28^ Moreover, self-efficacy mediates the relationship between parental expectations, academic stress, and performance, emphasizing its significance in academic outcomes. The correlation between self-efficacy and academic performance is particularly evident in vocational high school students, where self-efficacy significantly influences academic success. Overall, enhancing self-efficacy levels is vital for improving academic performance and student outcomes in various educational domains, highlighting the importance of fostering self-belief and confidence in learners. Therefore, the need to study the relationship between Academic stress on Student’s Psychological well-being, the following hypothesis is suggested.

Based on the above discussion the researchers proposed the following hypotheses:

H1.
There is a significant impact of academic stress on psychological well-being,

H2.
There is a significant impact of academic stress on social support.

H3.
There is a significant impact of academic stress on self-efficacy.

H4.
There is a significant impact of social support on psychological well-being,

H5.
There is a significant impact of self-efficacy on psychological well-being.

H6.
Social supports mediate the relationship between academic stress and psychological well-being.

**Figure 1.**
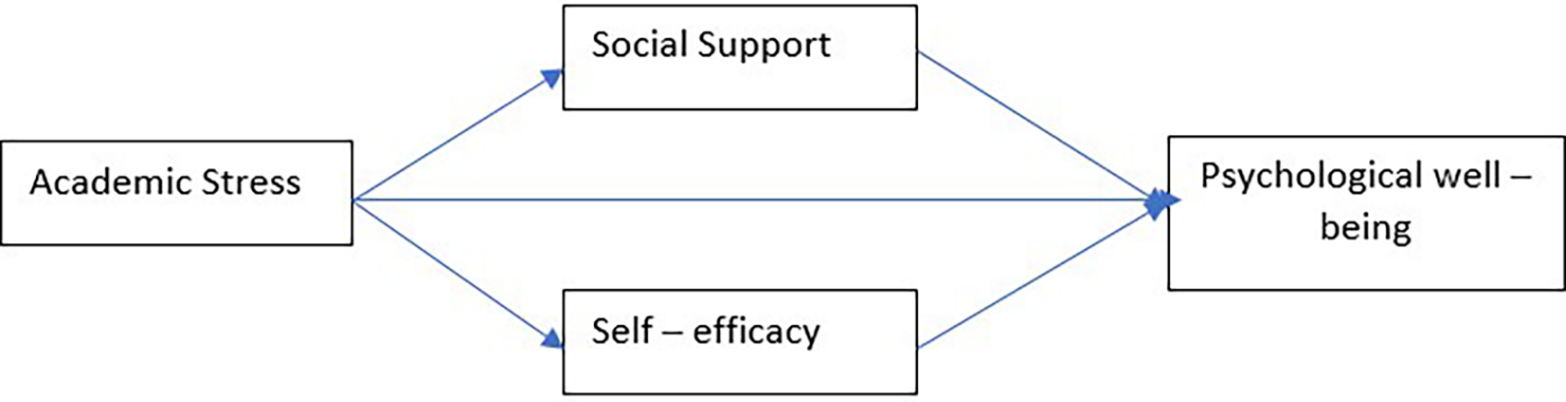
Research Model proposed by the authors

H7.
Self-efficacy mediates the relationship between academic stress and psychological well-being ( Figure 1).

## 3. Methods

### 3.1 Context of the study, population and sample

The context of the study was university students in Mogadishu – Somalia. Nearly 50,000 students are intended in these universities. These universities have fields of study at undergraduate, diploma and postgraduate levels. Faculties provided these universities include social science, computer technology, public health, business Economics and management sciences, sharia and law studies, education, engineering, medicine and surgery, Arts & humanities, agriculture & Environmental Sciences, languages, Islamic studies, geology, media & journalism, public admin and politics dentistry and postgraduate studies. The population of the study are target population from which the sample is actually selected or aggregation of study elements. ^29^ The study population are undergraduate students of these universities, its estimated around 30,000 students. The study included stratified sample technique. Stratified sample technique is type of sampling method in which the total population is divided into smaller groups or strata to complete the sampling process. ^30^ The study examined various factors related to academic stress and psychological well-being. The sample includes 663 students, with a gender distribution of 40.4% female and 59.6% male.

### 3.2 Research instrument and measures

A survey questionnaire was developed as the instrument for data collection. Items used for Academic Stress adapted by Ref. 31 to get students’ views of academic pressures and consists of 10 items, Psychological well-being Scale (PWS) adapted by Ref. 21 and consist of 18 items, Social Support Scale (SSS) adapted by Ref. 32 and 12 items, Self-Efficacy Scale (SES) adapted by Ref. 33 and consist of 10 items.

### 3.3 Data collection strategy

Data collected using adopted questionnaires distributed electronically, depending on the feasibility and preferences of participants. The questionnaire comprised validated measures assessing academic stress, psychological well-being, social support, and self-efficacy. Additionally, demographic information such as age, gender, academic major, and university affiliation collected to facilitate subgroup analyses. The data collected from May 15 to Jun 3 2024.

### 3.4 Data analysis

Descriptive statistics used to characterize the sample and examine the distribution of key variables. Structural equation modeling was used to examine mediating analysis of social support, self-efficacy in the relationship between academic stress, and psychological well-being. AMOS – SPSS 22 was used to estimate the measurement model, structural model as well as mediating analysis based on a 5000-bootstrap method of social support, self-efficacy in the relationship between academic stress, and psychological well-being.

### 3.5 Ethical consideration

Prior to data collection, ethical approval was received from Mogadishu University institutional review board (IRB) in Mogadishu, Somalia (Date:15 – 05 – 2024, protocol number: MUIRB/09/24/001). Informed consent was obtained from all students. The students were informed that the research would be conducted following the principle of confidentiality and that all information obtained would be kept confidential. We obtained informed consent from all participants, according to the IRB’s guidelines. Each of the 663 participants reviewed and signed the consent form before participating in the study. We confirm that all participants adhered to the signed consent form.

## 4. Data analysis and results

### 4.1 Profile data

A total of 663 sample size was achieved, the demographic profile of respondents for this study showed that female respondents made up 40.4%, of the sample while male made up 59.6% and mostly aged 18 to 21 years 67.1%. The distribution between public (52.6%) and private (47.4%) universities is nearly even. Most students are in their first three years of study, with Year One students representing the largest group 36.2%, while there are few students in Year Five (0.9%) and Year Six (1.2%) ( Table 1).

**Table 1.** Demographic profile data.

		Frequency	Percentage (%)
Gender	Female	268	40.4%
	Male	395	59.6%
Age	18 to 21 years	445	67.1%
	22 to 31 years	212	32.0%
	32 to 41 years	6	0.9%
University sector	Public	349	52.6%
	Private	314	47.4%
Study year	Year One	240	36.2%
	Year Two	152	22.9%
	Year Three	178	26.8%
	Year Four	79	11.9%
	Year Five	6	0.9%
	Year Six	8	1.2%

Note: Primary data collected by authors 2024.

**Measurement model**

The results indicate strong reliability and validity across the constructs, as evidenced by the factor loadings, squared multiple correlations (SMC), and Cronbach’s alpha values. The factor loadings range from **0.595 to 0.855**, with most indicators exceeding the acceptable threshold of 0.6, demonstrating significant contributions to their respective constructs and indicating good convergent validity. ^34^ The SMC values range from **0.354 to 0.731**, indicating that the latent constructs explain a reasonable proportion of the variance in their indicators. Cronbach’s alpha values are consistently above the recommended threshold of 0.7, ^35^ ranging from **0.755 to 0.906**, confirming good internal consistency for all constructs. Similarly, the composite reliability (CR) values for all constructs are above the recommended threshold, suggesting good internal consistency. However, the average AVE values for the academic stress (0.399) and psychological well-being (0.487) constructs are below the recommended threshold of 0.50, indicating that these constructs may not be capturing a sufficient amount of variance in their respective indicators. The correlation matrix reveals moderate to strong relationships between the constructs, with the highest correlation observed between self-efficacy factor and psychological well-being (0.814), suggesting a strong positive association between these two variables. Overall, the results provide insights into the psychometric properties of the measured constructs and their interrelationships within the study context. The discriminant validity was achieved. The result presented in Tables 2 and 3.

**Table 2.** Factor loading, Composite reliability (CR) and average variance extracted (AVE).

	Factor loading	SMC	Alpha
Academic stress			0.755
AS1	0.604	0.365	
AS2	0.704	0.496	
AS3	0.614	0.377	
AS4	0.597	0.356	
AS5	0.632	0.399	
Social support factor			0.906
SO1	0.851	0.724	
SO2	0.855	0.731	
SO3	0.835	0.697	
SO4	0.819	0.671	
Self-efficacy factor			0.843
SES1	0.787	0.619	
SES2	0.790	0.624	
SEs3	0.706	0.498	
SES4	0.744	0.554	
Psychology well-being			0.821
PWB1	0.740	0.548	
PWB2	0.782	0.612	
PWB3	0.595	0.354	
PWB4	0.674	0.454	
PWB5	0.683	0.466	

Note: Primary data collected by authors 2024.

SMC: Squared Multiple Correlation.

**Table 3.** Discriminant validity.

	CR	AVE	SEF	AS	SSF	PWB
**SES**	0.843	0.574	**0.758**			
**AS**	0.768	0.399	0.068	**0.631**		
**SSF**	0.906	0.706	0.516	0.076	**0.840**	
**PWB**	0.825	0.487	0.814	0.189	0.430	**0.698**

Note: Primary data collected by authors 2024.

Composite reliability (CR); average variance extracted (AVE); Academic stress (AS); psychological well-being (PWB); social support factor (SSF), self – efficacy scale (SES).

**Structural model**

The structural model illustrates the relationships among the constructs in the proposed study framework. H1 indicates that academic stress significantly negative impacts students’ psychological well-being. The findings revealed that academic stress has significant negative impact (total effect) on psychological well-being ( *β* = 0.870, t = 3.287, p < 0.001). Hence, H1 supported. However, higher academic stress corresponds to lower psychological well-being, it could indicate that manageable levels of academic stress serve as a motivator or enhance students' resilience, ultimately contributing positively to their psychological well-being. H2 reveals a weak but significant positive relationship between social support factors (SSF) and academic stress (AS) (β = 0.088, t = 2.314, p = 0.021). H2 supported, similarly, H3 demonstrates a weak positive relationship between social support factors (SSF) and psychological well-being (PWB) (β = 0.099, t = 5.278, p < 0.0001). Finally, H4 highlights a strong positive relationship between self-efficacy scale (SES) and psychological well-being (PWB) (β = 0.646, t = 24.278, p < 0.0001). The result presents in the Table 4.

**Table 4.** Hypotheses test.

	Path coefficient	T statistics	P values
H1: AS➔PWB	0.870	3.287	0.001
H2: SSF ➔AS	0.088	2.314	0.021
H3: SSF➔PWB	0.099	5.278	0.000
H4: SES➔PWB	0.646	24.278	0.000

Note: Primary data collected by authors 2024.

Academic stress (AS); psychological well-being (PWB); social support factor (SSF), self-efficacy scale (SES).

**Mediation analysis**

**Figure 2.**
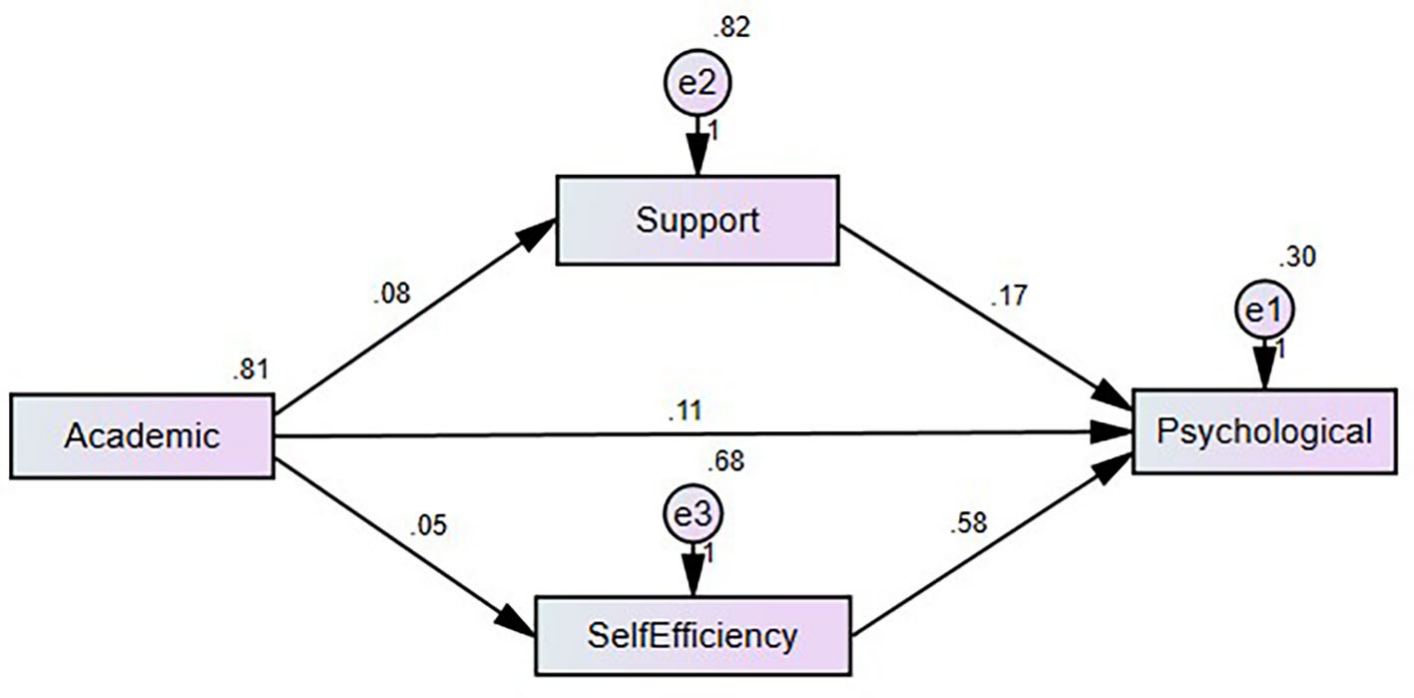
Mediation model proposed by the authors.

The results demonstrate the mediating roles of social support factors (SSF) and self-efficacy scale (SES) in the relationship between academic stress (AS) and psychological well-being (PWB). For the AS → SSF → PWB relationship, academic stress has a significant direct effect on PWB (β = 0.117), and the indirect effect through SSF is small but statistically significant (β = 0.012, p = 0.041). The confidence interval [0.002, 0.024] does not include zero, indicating reliable partial mediation. This suggests that social support factors play an important role in buffering the effects of academic stress on psychological well-being. In contrast, for the AS → SEF → PWB relationship, academic stress has a weaker direct effect on PWB (β = 0.088). Although the indirect effect through SEF is significant (β = 0.057, p = 0.068), the confidence interval [0.006, 0.111] includes zero, indicating no substantial mediation. This implies that while self-efficacy is influenced by academic stress, it does not meaningfully mediate the connection between academic stress and psychological well-being. These findings highlight the critical role of social support in mitigating the negative impacts of academic stress, whereas self-efficacy may have a limited mediating effect. Mediation analysis summary is presented in Table 5 and Figure 2.

**Table 5.** Mediating role social support and self-efficacy.

Relationship	Direct effect	Indirect effect	Confidence interval		P-value	Conclusion
			Lower Bounded	Upper Bound		
AS->SSF->PWB	0.117	0.012	−0.002	0.024	0.041	Partial Mediation
AS->SEF->PWB	0.088	0.057	0.061	0.111	0.068	No Mediation

Note: Primary data collected by authors 2024.

**Table 6.** Summary of the hypotheses.

Hypotheses	Finding
**H1.** There is a significant impact of academic stress on psychological well-being	Supported
**H2.** There is a significant impact of academic stress on social support.	Supported
**H3.** There is a significant impact of academic stress on self-efficacy.	Supported
**H4.** There is a significant impact of social support on psychological well-being.	Supported
**H5.** Social support mediates the relationship between academic stress and psychological well-being.	Partial Mediation
**H6.** Self-efficacy mediates the relationship between academic stress and psychological well-being.	No Mediation

Note: Primary data collected by authors 2024.

**Evaluation model fit**

The model evaluation results indicate a good fit of the model to the data. The relative chi-square (2.537) is within the recommended threshold of less than 5, and all the fit indices, including the Goodness of Fit Index (0.948), Adjusted Goodness of Fit Index (0.931), Comparative Fit Index (0.962), Incremental Fit Index (0.963), Normed Fit Index (0.940), Tucker-Lewis Index (0.955), and the Root Mean Square Error of Approximation (0.048), meet or exceed their respective recommended thresholds. Additionally, the Standardized Root Mean Square Residual (0.0369) is within the recommended limit of less than 0.05, ^36, 37^ further supporting the good fit of the model ( Table 7).

**Table 7.** Model fit indices.

	Recommended	Result
Relative Chi-Sq	Chi<5	2.537
Goodness fit index (GFI)	GFI≥.90	0.948
Adjusted goodness fit index (AGFI)	AGFI≥.90	0.931
Comparative fit index (CFI)	CFI≥.90	0.962
Incremental fit index (IFI)	IFI≥.90	0.963
Normed fit index (NFI)	NFI≥.90	0.940
Tucker-Lewis Index	TFI≥.90	0.955
Root Mean Square Error of Approximation (RMSEA)	RMSEA≤0.08	0.048
Standard RMR	SRMR≤0.05	0.037

Note: Primary data collected by authors 2024.

## 5. Discussion

The study is set out to establish the relationship between academic stress (AS), student’s psychological well-being (PWB), social support factor (SSF) and self-efficacy scale (SES). The study found a significant impact of academic stress on student psychological well-being among public and universities in Mogadishu - Somalia. The outcomes affirm this recommendation are steady with the discoveries of the past examinations where the positive relationship between academic stress (AS) and psychological well-being (PWB) outcomes was found. ^24, 25, 38^ This relationship indicates that the pressures of academic demands, such as exams and assignments, can lead to negative psychological outcomes, including anxiety and decreased life satisfaction. The findings align with the Transactional Model of Stress and Coping, which suggests that stress occurs when individuals perceive demands exceeding their coping abilities. The unique socio-cultural and educational challenges in Somalia, such as limited resources and high expectations, may intensify the effects of academic stress, emphasizing the need for support systems and interventions to help students manage stress and improve their psychological well-being.

The study found insignificant impact of academic stress on social support factor, suggesting that academic stress does not significantly alter the level of social support students perceive or receive. This result may indicate that social support, whether from peers, family, or faculty, remains stable despite fluctuations in academic stress levels. Previous research has shown that while social support can buffer the effects of stress, the presence of stress does not necessarily reduce the availability or perception of social support. ^24, 25, 38^

This stability might be due to cultural or social norms that encourage strong support networks, regardless of individual stress levels. Additionally, social support might be more influenced by factors such as personal relationships and community dynamics rather than academic pressures.

The study found that academic stress had an insignificant impact on self-efficacy among students in university students in Mogadishu. This suggests that the students’ belief in their ability to manage and succeed in academic tasks was not significantly affected by the levels of academic stress they experienced. This finding is inconsistent with research indicating that self-efficacy is often a stable trait influenced more by long-term experiences and personal characteristics than by situational stress. ^26, 39^ Self-efficacy may be bolstered by intrinsic factors such as past successes, individual resilience, and consistent encouragement from mentors and peers, which help maintain students’ confidence in their abilities despite academic pressures.

The study found that social support significantly impacts psychological well-being (PWB) among students in university students in Mogadishu. This means that students who receive higher levels of social support from peers, family, and faculty tend to have better psychological well-being, experiencing less anxiety and higher life satisfaction. This finding aligns with previous research which indicates that social support acts as a buffer against stress and promotes mental health by providing emotional comfort, practical assistance, and a sense of belonging. ^32^^–^^34^ The presence of a strong support network can mitigate the adverse effects of stress and enhance overall well-being by improving coping mechanisms and providing resources for managing academic challenges.

The study found that self-efficacy significantly impacts psychological well-being (PWB) among students in university students in Mogadishu. This indicates that students who have a strong belief in their ability to manage and succeed in academic tasks tend to have better psychological well-being, with lower levels of anxiety and higher life satisfaction. This finding is supported by previous research showing that self-efficacy enhances mental health by promoting resilience, reducing stress, and encouraging proactive coping strategies. ^41, 43, 44^ High self-efficacy helps students to perceive challenges as manageable and to maintain a positive outlook, which contributes to better psychological well-being.

Finally, the results of this research offer important empirical insight into the indirect effect on academic stress on psychological well-being through mediation of social support factor. The finding shows that social support partially mediation the relationship between academic stress and psychological well-being. This consistent with the previous finding which have found the significant mediating role of social support ^32, 45, 46^ which suggests that social support can mitigate the harmful effects of stress by providing emotional comfort, practical help, and a sense of belonging, thus enhancing overall psychological well-being. On other hand, the study found that self-efficacy does not mediate the relationship between academic stress and psychological well-being (PWB) among students in Mogadishu’s universities. This means that while self-efficacy directly contributes to better psychological well-being, it does not significantly alter the impact of academic stress on well-being. This finding contrasts with some studies that suggest self-efficacy can buffer stress impacts ^32^ indicating that in this context, other factors may play a more significant role in mediating the relationship between academic stress and psychological well-being.

## 6. Conclusion

In conclusion, the study reveals several key findings about the relationships between academic stress, psychological well-being (PWB), social support, and self-efficacy among students in Mogadishu’s universities. Academic stress significantly impacts students’ psychological well-being, highlighting the need for interventions to manage academic pressures. Social support plays a critical role in partially mediating this relationship, buffering some of the negative effects of academic stress on mental health. In contrast, self-efficacy, while positively contributing to psychological well-being, does not mediate the relationship between academic stress and psychological well-being (PWB). These insights underscore the importance of enhancing social support networks and developing targeted strategies to improve student resilience and mental health in the face of academic challenges.

## 7. Implication

Universities should prioritize the development and enhancement of student support services, including counseling and mental health resources, to help students manage academic stress and improve psychological well-being. More broadly, the study contributes to the development of studies linking academic stress, social support, self -efficacy and psychological well-being and strengthens the relationship evidence in the literature that social support contributes to psychological well -being. Institutions should encourage the formation of strong social networks through peer mentoring, student organizations, and community-building activities, as social support is crucial in buffering the negative effects of academic stress. Although self-efficacy does not mediate the stress-well-being relationship, enhancing students’ confidence in their abilities through workshops, positive feedback, and opportunities for skill development can directly improve their psychological well-being. Additionally, the study adds knowledge to studies seeking to understand the factors that influence psychological well-being. Thus, the findings suggest that universities should develop and implement stress management programs that equip students with strategies to cope with academic demands, thus improving their psychological well-being. Institutions should promote social support systems through peer mentoring, counseling services, and community-building activities to buffer students against the negative effects of academic stress. Institutions should promote social support systems through peer mentoring, counseling services, and community-building activities to buffer students against the negative effects of academic stress. Educational policies should prioritize mental health by incorporating stress management and support systems into the academic environment, ensuring holistic student development. Finally, it’s important that educational policymakers should integrate mental health education and stress management strategies into the curriculum, ensuring students are equipped with the tools to handle academic pressures effectively.

## 8. Research limitations and further research

The study’s limitations include its cross-sectional design, which limits causal inference, and the reliance on self-reported measures, which may introduce biases. Additionally, the findings are context-specific to Mogadishu, Somalia, and may not be generalizable to other settings. The research also focused narrowly on social support and self-efficacy as mediators, potentially overlooking other influential factors. Future studies should explore other potential mediators and moderators in the relationship between academic stress and psychological well-being, such as coping styles, resilience, and environmental factors, to develop a more comprehensive understanding and targeted interventions.

## Data Availability

Figshare: Mediating role of Social Support and Self-efficacy on Academic stress and Student’s Psychological well-being among University Students in Mogadishu -Somalia. https://doi.org/10.6084/m9.figshare.26820745 ^47^ The project contains the following data:
- Academic stress and Psychological well being.xlsx - Pschological Well being Mediating Role Updated.sav Figshare: Mediating role of Social Support and Self-efficacy on Academic stress and Student’s Psychological well-being among university students in Mogadishu-Somalia. https://doi.org/10.6084/m9.figshare.26820745 ^47^ The project contains the following data:
- Analyzing the results and calculating p-value using 5000 Bootstrap.docx - Measurement model.jpg Data are available under the terms of the Creative Commons Zero “No rights reserved” data waiver (CC0 1.0 Public domain dedication).
