# Supplementary material for: Mediating role of Social Support and Self-efficacy on Academic stress and Student’s Psychological well-being among University Students in Mogadishu -Somalia
Source: F1000Res. 2025 May 30;13:1108. Originally published 2024 Sep 30. [Version 3] doi: 10.12688/f1000research.155275.3 (PMC12141996; doi:10.12688/f1000research.155275.3)
Supplement: Peer review history

# Reviewer report: Reviewer response for version 3

**DOI:** 10.5256/f1000research.182948.r389314

**Published:** 2025-06-05

**Authors:** Hengki Yandri

**Affiliations:** 1 Kerinci Regency, Jambi, Indonesia

**Areas for Improvement (Reasons for Revision):**

**Methodological Detail:** The methods section requires more detail to allow for full replication (e.g., psychometric properties of instruments, more detailed data collection procedures, justification for sample size).

**Literature Integration & Originality: **The introduction and discussion would benefit from deeper integration with the existing literature and a stronger explanation of the study's original contribution, especially within its geographical context.

**Clarification of Results & Interpretation:** Some parts of the discussion could be clearer in interpreting the results, especially the implications of the mediating effects.

**Stronger Limitations:** The limitations section needs to be expanded to include methodological limitations and their implications for the findings.

**Citing Current Literature: **There is an opportunity to incorporate more recent studies to demonstrate a comprehensive understanding of the field.

 Overall, this research has a solid foundation and contributes to the literature. With the suggested revisions, the scientific quality and clarity of presentation of this article will significantly improve, making it suitable for indexing in a reputable journal.

Is the work clearly and accurately presented and does it cite the current literature?

Yes

If applicable, is the statistical analysis and its interpretation appropriate?

Yes

Are all the source data underlying the results available to ensure full reproducibility?

Yes

Is the study design appropriate and is the work technically sound?

Yes

Are the conclusions drawn adequately supported by the results?

Yes

Are sufficient details of methods and analysis provided to allow replication by others?

Yes

Reviewer Expertise:

Spiritual Counseling, Educational Psychology, Counseling, and Mental Health

I confirm that I have read this submission and believe that I have an appropriate level of expertise to confirm that it is of an acceptable scientific standard, however I have significant reservations, as outlined above.

# Reviewer report: Reviewer response for version 3

**DOI:** 10.5256/f1000research.182948.r388700

**Published:** 2025-06-03

**Authors:** Zartashia Kynat Javaid

**Affiliations:** 1 Government College University Faisalabad, Faisalabad, Punjab, Pakistan

Amendments are satisfactory.

Is the work clearly and accurately presented and does it cite the current literature?

Yes

If applicable, is the statistical analysis and its interpretation appropriate?

Yes

Are all the source data underlying the results available to ensure full reproducibility?

Yes

Is the study design appropriate and is the work technically sound?

Yes

Are the conclusions drawn adequately supported by the results?

Yes

Are sufficient details of methods and analysis provided to allow replication by others?

Yes

Reviewer Expertise:

Applied Psychology

I confirm that I have read this submission and believe that I have an appropriate level of expertise to confirm that it is of an acceptable scientific standard.

# Reviewer report: Reviewer response for version 2

**DOI:** 10.5256/f1000research.177201.r379969

**Published:** 2025-05-21

**Authors:** Zartashia Kynat Javaid

**Affiliations:** 1 Government College University Faisalabad, Faisalabad, Punjab, Pakistan

I appreciate your efforts to carry out this work. Authors have addressed the concerns and revision is significantly improved. I have one suggestion for the sake of further improvements of sections and coherence. Introduction section must include atleast definitions of all study variables. You should reorganize introduction and literature review part to align well. I recommend consulting this systematic review covering areas and literature on psychological wellbeing throwing light on outcome variable.(Refer to 1 )

Is the work clearly and accurately presented and does it cite the current literature?

Yes

If applicable, is the statistical analysis and its interpretation appropriate?

Yes

Are all the source data underlying the results available to ensure full reproducibility?

Yes

Is the study design appropriate and is the work technically sound?

Yes

Are the conclusions drawn adequately supported by the results?

Yes

Are sufficient details of methods and analysis provided to allow replication by others?

Yes

Reviewer Expertise:

Applied Psychology

I confirm that I have read this submission and believe that I have an appropriate level of expertise to confirm that it is of an acceptable scientific standard, however I have significant reservations, as outlined above.

## References

[1] Javaid, Z. K., Batool, H., Fatima, N., Muneer, A., & Azam, S. (2024). Eudaimonic well-being among young adults: A systematic review of influencing factors and outcomes. Journal of Policy Research, 10(2), 46-57.

# Reviewer report: Reviewer response for version 1

**DOI:** 10.5256/f1000research.170424.r353385

**Published:** 2025-01-10

**Authors:** Nguyen Tan Dat

**Affiliations:** 1 Can Tho University of Medicine and Pharmacy, An Khánh, Vietnam

Overall, the paper examines the impact of academic stress on psychological well-being among university students in Mogadishu, Somalia, exploring the mediating roles of social support and self-efficacy. The results indicate that social support partially mediates the relationship between academic stress and well-being, while self-efficacy does not. The study's use of robust methodology, including stratified sampling and structural equation modeling, and its practical implications for improving student mental health through social support, are key strengths.

 However, I believe that a significant rewrite and thorough English editing are necessary to enhance the quality of the article for submission

**Introduction**

1. Could the authors please provide the citation for this sentence in the second paragraph of the Introduction Introduction, paragraph 2: *“Also, other factors contributing to academic stress include self-inflicted stress, parental expectations, academic queries, lack of time for revision, low parental education levels, and poor exam grades.”*
2. I suggest the authors use only 'self-efficacy' for consistency throughout the paper. In my opinion, 'self-efficacy' is the more widely recognized and commonly used term.
3. It seems that the second citation is wrong: The paper name is supposed to be “Academic Stress Among University Students: A Quantitative Study of Generation Y and Z’s Perception”. “SOCIAL SCIENCES & HUMANITIES:” is part of the journal name.
4. I think it would be more beneficial to shorten the 2nd, 3rd, 4th,, 5th, 6th paragraph in the introduction because some ideas are repeated. For example this paragraph: *“Academic stress arises from the inability to cope with academic related tasks, leading to negative impacts such as smoking behavior, mental health issues, poor sleep quality, depression, insomnia, substance addiction, self-harm, and suicidal ideation. Also, other factors contributing to academic stress include self-inflicted stress, parental expectations, academic queries, lack of time for revision, low parental education levels, and poor exam grades.”*  And this paragraph *“Academic stress is the product of a combination of academic-related demands that exceed the adaptive resources available to an individual,2 Researchers agree that students face common academic stressors, such as family-pressures, scholarship demands, financial burdens, competition in class, examination, time-management and course-related stress.2 According to Ref. 3 the main cause of academic stress are academic results, admissions, students transfer, exams, load of extra classes, social comparison, exams stress, expectations teachers and parents.”* Both paragraphs talk about the cause of academic stress
5. I also feel that this sentence: *“Academic stress arises from the inability to cope with academic related tasks, leading to negative impacts such as smoking behavior, mental health issues, poor sleep quality, depression, insomnia, substance addiction, self-harm, and suicidal ideation.” *and this sentence: *“The most common psychological consequences associated with academic stress include depression, anxiety, substance abuse, and suicide ideation.7” *are similar.
6. I have taken a look at the source of citation for this sentence “ *“Additionally, coping strategies like Academic Psychological capital and self-efficacy play a crucial role in mitigating the effects of academic stress on psychological well-being.”* and could not find any information about how self-efficacy and academic psychological capital mitigates the effect of academic stress on psychological well-being. Can the authors highlight it? This is the paper that I found for your citation. Ref (1)
7. I don’t understand what this sentence want to say: In the context of academic stress, this theory suggests that the students have the ability to cope with high self-efficacy are more likely to effectively cope with stressors and maintain their psychological well-being.
8. Citation number 9 format is different than others.
9. The authors lack citation for Social Cognitive Theory.
10. Why is coping mechanisms capitalized, and why theories is plural in this sentence *“In Coping Mechanisms, the theories emphasize the importance of coping strategies in managing stress.”* ? Do you mean to talk about “The Transactional Model of Stress and Coping”and “Social Cognitive Theory”?
11. Additionally, when the authors wrote these two paragraphs under Social Cognitive Theory, it seems like these paragraphs were talking about the theory. But I think these paragraphs are talking about common themes of both “The Transactional Model of Stress and Coping”and “Social Cognitive Theory” theories:  

*“In the context of the impact of academic stress on students’ psychological well-being, the Transactional Theory of Stress and Coping and the Social Cognitive Theory focus on the following. Both theories highlight the importance of cognitive appraisal in how individuals perceive and respond to stress. Students’ cognitive evaluations of academic stressors, their perceived ability to cope (self-efficacy), and the availability of social support influence their stress experiences and psychological well-being.10”*

*“ In Coping Mechanisms, the theories emphasize the importance of coping strategies in managing stress. Student performance related to cognitive appraisals and self-efficacy beliefs play an important role in mediating the relationship between academic stress and psychological well-being. Effective strategies, supported by social resources, help students manage academic stress and maintain well-being.9,10,15”*

 So I suggest the authors add another title for these 2 paragraphs.

1. Why also is the subject in this sentence? *“Also has emerged as a significant mental health that affecting various groups of students.”*
2. Don’t break this sentence into another paragraph: A study found a strong correlation (r=0.582) between academic stress and psychological well-being among university students, indicating that higher stress levels correspond to lower well-being.
3. Also, I feel these paragraphs titled “Academic Stress and Psychological Well-being” is a repeat of the start of the Introduction. I suggest the author omitted or combined these paragraph together with those paragraphs at the start of the Introduction.
4. I noticed that several common terms are inconsistently capitalized (e.g., 'Academic Psychological capital,' 'Health Sciences'). In academic writing, only proper nouns, titles, or the first words of sentences should be capitalized. I recommend reviewing the manuscript for unnecessary capitalization and ensuring it follows standard conventions for consistency and clarity.
5. I feel the Introduction is too long, it would be beneficial to be shorter

**Methods**

1.  I don't think it is necessary to go into such depth about the targeted universities (e.g., the number of students, the included faculties, etc.).
2.  There are three things wrong with this part of the sentence *“Self-Efficiency Scale (SSS) adapted by Ref. 35 and consist of 10 items.”*: First, it is self-efficacy not self-efficiency. Second, if the scale is called Self-efficacy scale, is should be abbreviated as SES not SSS. Third, The scale in Reference 35 is called “General Self-Efficacy Scale”, not Self-efficacy Scale
3. The authors did not mention which software used for data analysis.

**Results**

1. For very small p-values, avoid reporting them as 0.000; instead, use p < 0.001.
2. Please check carefully for grammar issues( *The result presents in the Table 4*->The result is presented in Table 4., partially mediation-> partial mediation)
3. In Table 4, the meaning of the symbol *'H1: AS* *à* *PWB*' is unclear.
4. I noticed that the author sometimes reports results with four decimal places (e.g., 0.0075) and other times with three decimal places (e.g., 3.524). To ensure consistency and clarity, I recommend using three decimal places consistently throughout the paper.
5. Please ensure that each abbreviation is defined or explained in every table for clarity.
6. Please cite Figure 2 in the Results, I also suggest changing Figure 2 name to reflect the mediation model.

**Discussion**

 25. I noticed that the abbreviation 'PWB' for 'psychological well-being' is introduced suddenly in the Discussion section, despite the full term being used throughout the paper. For consistency and clarity, it would be helpful to either introduce the abbreviation earlier in the text or continue using the full term throughout the manuscript: The study found that social support significantly impacts psychological well-being (PWB)

**Conclusion**

 26. Please put the conclusion paragraph at the end instead of before 6.1 Implications and 6.2 Limitations.

 27. I believe the order of the sections could be improved for better clarity and logical flow. In the current structure, '6.1 Implications' and '6.2 Limitations and Further Research' are listed as sub-sections under '6. Conclusion,' which may not be ideal. Typically, implications and limitations are treated as separate sections rather than sub-sections of the conclusion. I suggest considering reorganizing the sections, perhaps by placing 'Implications' and 'Limitations and Further Research' as standalone sections after the conclusion for a clearer structure.

 28. I noticed that the authors have cited their own preprint (citation 42). While self-citation is not inherently unethical, it may be worth considering if this citation is necessary and appropriate, especially in light of the potential for bias. It might be helpful to ensure that all citations are relevant and contribute to the academic discourse in a transparent manner.

 29. The paper investigates both public and private universities, but the results section does not address why data was collected from both types of institutions or highlight any notable differences between them. Additionally, there seems to be no analysis exploring potential distinctions between public and private universities, despite the title emphasizing the inclusion of both. It would strengthen the paper to either explore these differences or provide a rationale for including both types of universities in the study..

 30. the keyword 'Public and Private Universities' might be too broad. It could be helpful to consider a more specific keyword like "university students"

Is the work clearly and accurately presented and does it cite the current literature?

Partly

If applicable, is the statistical analysis and its interpretation appropriate?

I cannot comment. A qualified statistician is required.

Are all the source data underlying the results available to ensure full reproducibility?

Yes

Is the study design appropriate and is the work technically sound?

Partly

Are the conclusions drawn adequately supported by the results?

Partly

Are sufficient details of methods and analysis provided to allow replication by others?

Partly

Reviewer Expertise:

Clinical psychology

I confirm that I have read this submission and believe that I have an appropriate level of expertise to state that I do not consider it to be of an acceptable scientific standard, for reasons outlined above.

## References

[1] : How Do School Counselors Advocate for Gifted and Intelligent Children in Junior High School?. *Bisma The Journal of Counseling* .2022;6(3) : 10.23887/bisma.v6i3.50671 329-334 10.23887/bisma.v6i3.50671

# Response

**Published:** 2025-01-18

**Authors:** Abdulkadir Jeilani

**Affiliations:** Computer Science & IT, Mogadishu University, Mogadishu, Banaadir, Somalia

We sincerely thank the reviewer for his insightful and constructive feedback, which has significantly contributed to improving the quality and clarity of our manuscript. We have carefully addressed each comment and made the suggested revisions as detailed below:

**Introduction**

1. **Comment:** Could the authors provide a citation for this sentence:
*“Also, other factors contributing to academic stress include self-inflicted stress, parental expectations, academic queries, lack of time for revision, low parental education levels, and poor exam grades.”*
**Response:** The citation for this statement has been included.
2. **Comment:** Use 'self-efficacy' consistently throughout the manuscript for clarity.
**Response:** We have replaced all instances of "self-efficiency" with "self-efficacy."
3. **Comment:** The second citation is incorrect; the paper title should be *"Academic Stress Among University Students: A Quantitative Study of Generation Y and Z’s Perception."*
**Response:** The incorrect citation has been corrected.
4. **Comment:** Consider shortening paragraphs 2–6 of the Introduction, as they contain repetitive ideas.
**Response:** We combined the content of these paragraphs into two concise paragraphs to avoid redundancy.
5. **Comment:** These sentences appear similar and repetitive:
*“Academic stress arises from the inability to cope with academic-related tasks, leading to negative impacts such as smoking behavior, mental health issues, poor sleep quality, depression, insomnia, substance addiction, self-harm, and suicidal ideation.”*
*“The most common psychological consequences associated with academic stress include depression, anxiety, substance abuse, and suicide ideation.”*
**Response:** The repetition has been removed, and the sentences have been streamlined for clarity.
6. **Comment:** Could the authors clarify how self-efficacy and academic psychological capital mitigate the effects of academic stress on psychological well-being?
**Response:** We reviewed the source and highlighted the relevant information to address this query.
7. **Comment:** This sentence is unclear:
*“In the context of academic stress, this theory suggests that the students have the ability to cope with high self-efficacy are more likely to effectively cope with stressors and maintain their psychological well-being.”*
**Response:** The sentence has been revised for clarity and grammatical accuracy.
8. **Comment:** Citation number 9 is formatted differently from others.
**Response:** The formatting inconsistency has been corrected.
9. **Comment:** Provide a citation for Social Cognitive Theory.
**Response:** We have included the appropriate citation for Social Cognitive Theory.

**Literature Review**

1. **Comment:** Why are “Coping Mechanisms” and “Theories” capitalized?
**Response:** These terms are no longer capitalized for consistency with academic conventions.
2. **Comment:** The paragraphs under *Social Cognitive Theory* appear to address common themes of multiple theories, not just Social Cognitive Theory. Consider adding a new title.
**Response:** A new title has been added to appropriately categorize these paragraphs.
3. **Comment:** This sentence is unclear:
*“Also has emerged as a significant mental health that affecting various groups of students.”*
**Response:** The sentence has been revised for clarity.
4. **Comment:** Avoid breaking this sentence into a new paragraph:
*“A study found a strong correlation (r=0.582) between academic stress and psychological well-being among university students, indicating that higher stress levels correspond to lower well-being.”*
**Response:** The sentence has been kept intact within the paragraph for continuity.
5. **Comment:** The *Academic Stress and Psychological Well-being* section is repetitive. Consider merging or omitting parts that overlap with the Introduction.
**Response:** The section has been merged with the relevant parts of the Introduction for conciseness.
6. **Comment:** Terms like 'Academic Psychological Capital' and 'Health Sciences' are inconsistently capitalized.
**Response:** We reviewed the manuscript to ensure capitalization consistency.

**Methods**

1. **Comment:** Details about targeted universities (e.g., the number of students, and included faculties) may be unnecessary.
**Response:** The description has been shortened to focus on relevant details.
2. **Comment:** There are three issues with this sentence:
*“Self-Efficiency Scale (SSS) adapted by Ref. 35 and consists of 10 items.”*
  - It should be "Self-efficacy," not "Self-efficiency."
  - The abbreviation should be "SES," not "SSS."
  - The correct name of the scale is the *General Self-Efficacy Scale.*
**Response:** All corrections have been made.
3. **Comment:** The software used for data analysis is not mentioned.
**Response:** The software has been specified in the Methods section.

**Results**

1. **Comment:** Report small p-values as *p < 0.001* instead of *0.000.*
**Response:** This has been corrected throughout the Results section.
2. **Comment:** Ensure grammatical accuracy (e.g., "The result presents in Table 4" → "The result is presented in Table 4").
**Response:** Grammar issues have been corrected.
3. **Comment:** In Table 4, the symbol 'H1: ASàPWB' is unclear.
**Response:** The symbol has been clarified.
4. **Comment:** Use three decimal places consistently across results.
**Response:** All results now use three decimal places for consistency.
5. **Comment:** Define or explain abbreviations in every table.
**Response:** All abbreviations are now defined within the tables.
6. **Comment:** Cite Figure 2 in the Results section and rename it to reflect the mediation model.
**Response:** Figure 2 has been cited and renamed accordingly.

**Discussion and Conclusion**

1. **Comment:** Introduce the abbreviation *PWB* for "psychological well-being" earlier or use the full term consistently.
**Response:** We now use the full term consistently throughout the manuscript.
2. **Comment:** Move the conclusion paragraph to the end and treat *Implications* and *Limitations* as separate sections.
**Response:** The Conclusion has been moved to the end, and *Implications* and *Limitations* are now standalone sections.
3. **Comment:** Consider whether the self-citation (citation 42) is necessary.
**Response:** This self-citation was included as supplementary data per journal guidelines.
4. **Comment:** Address differences between public and private universities or provide a rationale for including both.
**Response:** The rationale has been clarified, and notable distinctions are highlighted.
5. **Comment:** Replace the broad keyword *Public and Private Universities* with a more specific term like *university students.*
**Response:** The keyword has been replaced with *university students.*

# Reviewer report: Reviewer response for version 1

**DOI:** 10.5256/f1000research.170424.r342011

**Published:** 2024-12-17

**Authors:** Somaya Shokry Mohammad Mahmoud

**Affiliations:** 1 Imam Abdulrahman Bin Faisal University, Dammam, Eastern Province, Saudi Arabia

*The mediating analysis showed that social support partially mediated the relationship between academic stress and psychological well-being (Indirect effect = 0.070, CI [-0.036, 0.197], p = 0.000),*

**Social Support**: The indirect effect (0.070) is **partially mediated**, but the confidence interval (CI [-0.036, 0.197]) includes zero. This creates a discrepancy because the p-value (p = 0.000) suggests significance.

*Conclusions*

*The findings suggest a significant positive impact of academic stress on psychological well-being*

 revise this statement. I think  there is a significant negative impact of academic stress on psychological well-being

 "Why is the gender factor included in Figure 1?"

*A study found a strong correlation (r=0.582) between academic stress and psychological well-being among university students, indicating that higher stress levels correspond to lower well-being. ^17^*

 Is the correlation positive or negative?

 The study design is appropriate for the research objectives, and the work is technically sound overall.

 The study provides many details that enable replication, particularly regarding the sample, constructs, and statistical analysis. To improve replicability, the following should be addressed:

- Specify the software and analytical procedures used.

Overall, the statistical analysis and its interpretation are appropriate and align with standard practices. Key areas for improvement include addressing the confidence interval discrepancy for social support mediation, acknowledging the low AVE values, and providing more details on effect sizes. These adjustments would further strengthen the robustness and clarity of the statistical conclusions.

 The study makes a commendable effort by sharing its source data on Figshare, ensuring transparency and partial reproducibility. However, to achieve **full reproducibility**, additional documentation and sharing of analysis scripts are needed. This would allow other researchers to replicate the study comprehensively and with minimal barriers.

 he conclusions are mostly supported by the results, with some exceptions:

- The confidence interval issue for social support mediation should be addressed to confirm this conclusion.
- The practical significance of the relationships should be better reflected.
- The low AVE values should be discussed to acknowledge potential measurement limitations.

Addressing these points would make the conclusions more robust and aligned with the study's results.

Is the work clearly and accurately presented and does it cite the current literature?

Yes

If applicable, is the statistical analysis and its interpretation appropriate?

Partly

Are all the source data underlying the results available to ensure full reproducibility?

Partly

Is the study design appropriate and is the work technically sound?

Yes

Are the conclusions drawn adequately supported by the results?

Partly

Are sufficient details of methods and analysis provided to allow replication by others?

Partly

Reviewer Expertise:

Educational Psycholgy

I confirm that I have read this submission and believe that I have an appropriate level of expertise to confirm that it is of an acceptable scientific standard, however I have significant reservations, as outlined above.

# Response

**Published:** 2024-12-20

**Authors:** Abdulkadir Jeilani

**Affiliations:** Computer Science & IT, Mogadishu University, Mogadishu, Banaadir, Somalia

We sincerely appreciate the time and effort the reviewer has dedicated to providing insightful and constructive feedback on our manuscript. Your comments have significantly enhanced the quality and clarity of our work.

 We are particularly grateful for your detailed observations, including the suggestions to address the confidence interval discrepancy, improve the discussion on AVE values, and provide additional details to strengthen replicability. These recommendations have been invaluable in refining our analysis and ensuring the robustness of our conclusions.

 Thank you for recognizing our study's strengths and highlighting areas for improvement. Your expertise and thoughtful input have guided us in making meaningful revisions, and we are confident that the manuscript is much stronger due to your valuable contributions.

 Here are the responses for each comment

**Social Support: Confidence Interval Discrepancy and Indirect Effect**

**Reviewer Comment:**

 The indirect effect (0.070) is partially mediated, but the confidence interval (CI [-0.036, 0.197]) includes zero. This creates a discrepancy because the p-value (p = 0.000) suggests significance.

**Response:**

 We appreciate the reviewer highlighting this discrepancy. The p-value indicates statistical significance, but the confidence interval suggests potential variability in the indirect effect. To address this:

- We resample bootstrap with 5,000 now the inconsistency is solved

**Conclusions: Nature of the Impact of Academic Stress**

**Reviewer Comment:**

 The findings suggest a significant positive impact of academic stress on psychological well-being. Revise this statement to indicate there is a significant negative impact.

**Response**:

 Thank you for pointing this out. The relationship between academic stress and psychological well-being is indeed negative, as supported by the correlation analysis and theoretical framework. We revised the conclusion to:

 "The findings suggest that academic stress has a significant negative impact on psychological well-being, partially mediated by social support."

**Gender Factor in Figure 1**

**Reviewer Comment:**

            Why is the gender factor included in Figure 1?

**Response**:

              Thank you, we excluded Gender from the research model.

**Correlation Between Academic Stress and Psychological Well-being**

**Reviewer Comment:**

 A study found a strong correlation (r = 0.582) between academic stress and psychological well-being. Is the correlation positive or negative?

**Response**:

 The correlation is negative, as higher academic stress corresponds to lower psychological well-being. This is explicitly stated in the discussion section to avoid any ambiguity.

**Replicability: Software and Analytical Procedures**

**Reviewer Comment:**

            Specify the software and analytical procedures used to improve replicability.

**Response:**

  We added the following details:

- The software used for analysis was SPSS (version 24) and AMOS (version 22).
- Analytical procedures such as path analysis, bootstrapping (5,000 samples), and structural equation modeling were applied to test direct and indirect effects.

**Practical Significance of Relationships**

**Reviewer Comment:**

            The practical significance of the relationships should be better reflected.

**Response:**

 The partial mediation by social support indicates the importance of strengthening support systems to mitigate the negative effects of academic stress on well-being.

**Low AVE Values**

**Reviewer Comment:**

               The low AVE values should be discussed to acknowledge potential measurement limitations.

**Response:**

 We acknowledge the low AVE values for some constructs (e.g., academic stress: 0.399). Noted as a limitation, indicating potential issues with convergent validity. We recommended revising measurement items in future studies to better capture the constructs.

**Data Sharing and Reproducibility**

**Reviewer Comment:**

 Additional documentation and sharing of analysis scripts are needed to achieve full reproducibility.

**Response:**

 We shared analysis scripts and detailed documentation alongside the dataset on Figshare. This will ensure that researchers can replicate the analysis comprehensively.

**Practical Recommendations**

**Reviewer Comment:**

             Address practical recommendations based on the findings.

**Response:**

 We emphasized the importance of social support systems and stress management interventions in educational institutions. These could include peer mentoring programs, counseling services, and workshops on effective coping mechanisms.
